# Insights into Calpain Activation and Rho-ROCK Signaling in Parkinson’s Disease and Aging

**DOI:** 10.3390/biomedicines12051074

**Published:** 2024-05-13

**Authors:** Amy Gathings, Vandana Zaman, Narendra L. Banik, Azizul Haque

**Affiliations:** 1 Department of Microbiology and Immunology, Medical University of South Carolina, 173 Ashley Avenue, Charleston, SC 29425, USA; gathinga@musc.edu (A.G.); baniknl@musc.edu (N.L.B.); 2 Department of Neurosurgery, Medical University of South Carolina, 96 Jonathan Lucas Street, Charleston, SC 29425, USA; zamanv@musc.edu; 3 Ralph H. Johnson Veterans Administration Medical Center, 109 Bee Street, Charleston, SC 29401, USA

**Keywords:** Parkinson’s disease, calpain, α-synuclein, Rho-ROCK, neurodegeneration, aging

## Abstract

Parkinson’s disease (PD), a progressive neurodegenerative disease, has no cure, and current therapies are not effective at halting disease progression. The disease affects mid-brain dopaminergic neurons and, subsequently, the spinal cord, contributing to many debilitating symptoms associated with PD. The GTP-binding protein, Rho, plays a significant role in the cellular pathology of PD. The downstream effector of Rho, Rho-associated kinase (ROCK), plays multiple functions, including microglial activation and induction of inflammatory responses. Activated microglia have been implicated in the pathology of many neurodegenerative diseases, including PD, that initiate inflammatory responses, leading to neuron death. Calpain expression and activity is increased following glial activation, which triggers the Rho-ROCK pathway and induces inflammatory T cell activation and migration as well as mediates toxic α-synuclein (α-syn) aggregation and neuron death, indicating a pivotal role for calpain in the inflammatory and degenerative processes in PD. Increased calpain activity and Rho-ROCK activation may represent a new mechanism for increased oxidative damage in aging. This review will summarize calpain activation and the role of the Rho-ROCK pathway in oxidative stress and α-syn aggregation, their influence on the neurodegenerative process in PD and aging, and possible strategies and research directions for therapeutic intervention.

## 1. Introduction

Parkinson’s disease (PD) is a neurodegenerative disease that affects motor function, causing symptoms such as tremors, stiffness, difficulty with walking and talking, and balance and coordination problems [1,2]. The non-motor symptoms, such as constipation, loss of smell, disturbed sleep, memory, and cognitive changes, are evident before the onset of motor symptoms [3,4]. The neurodegenerative changes in PD are not only limited to the brain, but they are also detected in the spinal cord [5] and enteric nervous system [6,7]. PD is primarily characterized by a loss of dopaminergic neurons in the substantia nigra (SN) pars compacta in the mid-brain [1,8,9]. Evidence suggests that along with the dopaminergic system, other neurotransmitter systems, such as noradrenergic and cholinergic, also play a role in onset and/or progression of PD. The dysfunction of the locus coeruleus (LC) noradrenergic neurotransmitter system is indicated by the prodromal symptoms of PD, such as sleep disturbance [10], and in the later stages, orthostatic hypotension and apathy [11]. In PD, the loss of SN neurons reduces dopamine levels in the striatum, resulting in an increase in acetylcholine release by the cholinergic interneurons, which alters striatal cholinergic signaling, activity, and connectivity. Thus, understanding the crosstalk between these neurotransmitter systems is crucial for comprehending the pathogenesis of PD.

Although the etiopathology of the sporadic form of PD is still unknown, the familial forms of PD have revealed various key players that may be involved in PD pathogenesis. Notably, over 200 PD-related genes have been identified [12]. Some of these mutations are alpha-synuclein (α-syn) gene (SNCA), parkin (PRKN), PTEN-induced kinase 1 (PINK1), Leucine rich-repeat kinase 2 (LRRK2), Vacuolar protein sorter-35 (VPS35), coiled-coil-helix-coiled-coil-helix domain containing 2 (CHCHD2), prosaposin (PSAP), and DJ-1 [13,14,15]. These genetic mutations have provided crucial insight into the mechanisms and pathways involved in PD, such as mitophagy, oxidative stress, vesicular/intracellular trafficking, defects in mitochondrial respiratory complex, and α-syn aggregation. Furthermore, these mutations have helped in developing animal models to study PD pathogenesis and develop therapeutic interventions [16]. Information from genetic mutation and diseased brain autopsies has revealed some key players and pathways involved in the disease process. Considering these cues, animal models are created using neurotoxins to investigate the sporadic forms of PD [17,18]. The most prevalent models are intraperitoneal (i.p.) injections of 1-methyl-4-phenyl-1,2,3,6-tetrahydropyridine (MPTP) in mice, sub-cutaneous (s.c.) injections of rotenone in rats, or lesion of dorsal striatum by intracerebral infusion of *6*-*hydroxydopamine* (6-OHDA) in rats. These toxin-induced models imitate PD pathogenesis by replicating the degeneration of dopaminergic neurons in the SN.

Neuroinflammation is one of the pathophysiological processes among several pathways implicated in neurodegeneration. Evidence from PD patient brain samples illustrates that activated microglia and reactive astrocytes are present in the brain [19,20]. Similarly, the animal models have also indicated the presence of these activated glial cells in the nigrostriatal pathways [21,22]. Additionally, the protein α-syn activates microglia, leading to pro-inflammatory responses in the brain. This activation occurs when α-syn binds to integrin CD11b, which then activates the downstream Rho-ROCK pathway [23]. A number of studies have suggested that the over-activation of calpain, which is a calcium-dependent non-lysosomal cysteine protease, may play a critical role in the onset and/or progression of several neurodegenerative diseases [21,24,25,26,27]. The over-activation of calpain may also affect the downstream Rho-ROCK pathway. In PD, the role of calpain in its pathogenesis is suggested because α-syn, one of the proteins cleaved or degraded by calpain, forms Lewy bodies (LBs) [28,29,30]. It is, therefore, likely that the dysfunction of calpain activity may contribute to the onset of PD, and regulating calpain activity could be a viable therapeutic approach to intervene in neurodegeneration. In animal models of PD, our group has shown over-expression of calpain in the SN dopaminergic neurons [21,24,26]; hence, preventing the over-activation of calpain using a specific inhibitor could be a potential treatment option. These findings also suggest that neuroinflammation plays a critical role in the disease process and highlight the need for further research to explore potential neuro-immune interactions with other cell types.

The pathophysiology of PD shows similarities with the changes that occur during the aging process at a cellular and molecular level. Observations from animal disease models indicate that the characteristic features related to PD, such as mitochondrial dysfunction and oxidative stress, chronic inflammation, dysfunction in autophagy leading to the formation and accumulation of protein aggregates, functional deficit, and cognitive dysfunctions, are common in aging [31]. These similarities in cellular changes in aging and PD indicate that aging could be a risk factor in PD. Moreover, PD is typically diagnosed in individuals above 60 years of age, which further supports the theory that age is a predominant risk factor in PD. This review will discuss calpain activation, α-syn aggregation, and formation of LBs in PD, and calpain’s role in the regulation of the Rho-ROCK pathway in PD, related dementia, and aging.

## 2. Parkinson’s Overview

PD is a neurodegenerative disease that causes movement disorder, dementia, sleep behavior disorder, pain, and other health-related issues [8,32,33]. PD can be identified by signs of tremor, stiffness, difficulty walking and talking, and instability in both balance and coordination. It is also a disease of the central nervous system (CNS), which consists of the brain and spinal cord [24,26,34]. The brain is categorized as nervous tissue and it responds to memory, thought processes, communication, emotion, movement, sensations, and responses [33,35]. The spinal cord sends motor commands from the brain to the peripheral body and relays sensory information from sensory organs to the brain. The spinal cord has two distinct pathways: the ascending and descending pathways [33,36]. The ascending pathway is where sensory information travels from the body to the spinal cord and the brain. The descending pathway is where motor signals from the brain are sent to lower motor neurons, where efferent neurons then lead to muscle movement. As mentioned above, PD is characterized by the loss of SN dopaminergic neurons. This leads to a lower level of dopamine in the target area, the caudate putamen/striatum, and consequent diminished motor function, leading to clinical features of the disease [1]. This loss of dopamine in the caudate contributes to movement disorder, since the function of this nucleus is to plan the execution of movement. The caudate also affects memory and cognition [37,38]. The cholinergic interneurons are the main source of acetylcholine in the striatum and are innervated by the SN pars compacta dopaminergic neurons [39]. Acetylcholine release in the striatum by cholinergic interneurons is known to modulate striatal dopamine release [40,41]. Along with the loss of SN dopaminergic neurons, the extensive loss of LC noradrenergic neurons is also detected in PD patients [31,42,43,44]. The noradrenergic neurotransmitter system typically participates in stress response, emotional memory, and control of motor, sensory, and autonomic functions [45,46]. Several lines of evidence suggest that the α-syn pathology and other degenerative changes develop in the LC neurons before affecting the dopaminergic neurons in the SN pars compacta [47]. These findings strongly indicate that LC noradrenergic neuronal loss plays a critical role in disease initiation, progression, and severity.

## 3. Lewy Bodies Are a Key Characteristic of PD

**Figure 1:**
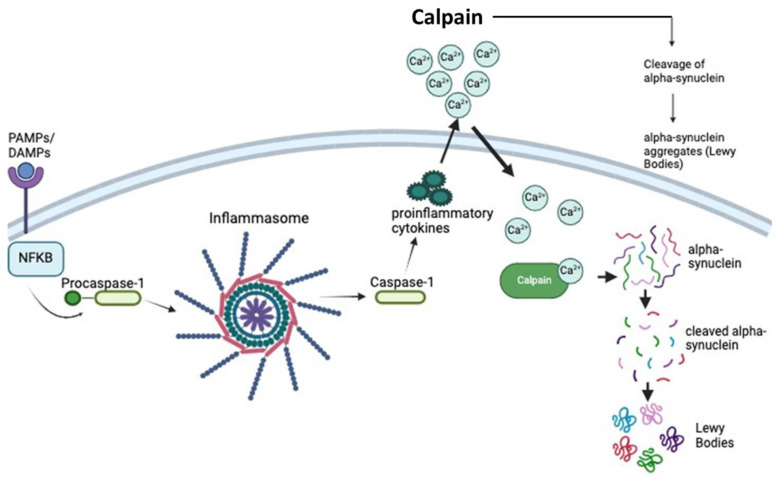
α-syn aggregation and formation of LBs. Initiation of inflammasome response may lead to activation of pro-inflammatory cytokines and calpains, triggering α-syn cleavage and aggregation, and LB formation. Arrows indicate the logical steps in the formation of LBs.

One of the major key players in PD is the aggregated form of α-syn, which plays multiple functions in the brain along with synaptic transmission and neurogenesis [48,49,50]. The presence of an aggregated form of α-syn contributes significantly to the formation of Lewy bodies (LBs), which are the pathological hallmark of PD [51,52,53]. LB inclusions are detected in the SN dopaminergic neurons in all cases of PD. Other brain areas in PD patients also show the presence of LB, such as LC, dorsal vagal nucleus, nucleus basalis of Meynert, cerebral cortex, olfactory bulb, thalamus, and hypothalamus [54,55,56]. LB production might begin with an inflammasome response to pathogen-associated molecular patterns (PAMPs) and damage-associated molecular patterns (DAMPs) [57]. This response leads to the conversion of procaspase-1 into caspase-1, which activates pro-inflammatory cytokines (Figure 1). This can also lead to the activation of calpain, which truncates α-syn. The truncated α-syn units bind with other truncated α-syn units, causing aggregates of the protein and LB formation. Not only can the presence of active proteases, such as calpain, cause cleavage of the protein at its C-terminal, but mutations in it are another culprit of cleavage for this particular α-syn protein [58,59,60]. These aggregates lead to penetration of neuronal membranes via toxic fibril structures, causing oxidative stress, calcium influx disruption, and neuronal apoptosis of dopamine-producing neurons in the nigrostriatal pathway [61,62].

α-syn is also involved in the activation of microglia and induces neuroinflammation. It interacts with integrin CD11b on microglia and this interaction triggers the activation of NADPH Oxidase 2 (NOX2), which leads to the production of ROS [23,63]. It has been shown that in PD patients, there is excessive ROS production, which may cause oxidative stress and damage to SN neurons [64,65,66]. Oxidative stress also activates ataxia–telangiectasia mutated kinases and ataxia–telangiectasia- and Rad3-related protein kinases. Together, these kinases deactivate oncoprotein MDM2 and activate p53 [67,68,69], ultimately leading to the release of pro-inflammatory cytokines. The mediators of the inflammation process are intracellular multiprotein complexes and inflammasomes, which contribute to neuron injury and death, as well as the activation of T cells.

Substantial intronic transcriptional changes in PD patients compared to healthy controls have been reported [70]. Although the exact cause of PD is unknown, several factors, such as genetic, environmental, and head trauma, are implicated in the disease process. Mutations in certain genes, such as *SNCA*, *PARK2*, *PARK7*, *PINK1*, and *LRRK2*, can lead to the development of PD [20,70,71,72,73]. The onset of neurodegenerative diseases may be influenced by a combination of environmental and genetic factors, including interactions between genes. Along with genetic mutations, various studies, such as genome-wide association studies (GWAS) and targeted single-nucleotide polymorphism (SNP) studies, have identified numerous loci and polymorphisms associated with neurodegenerative diseases [74,75,76,77]. In addition to mutations, another suspected genetic component associated with PD is the most abundant retrotransposons, SINE-VNTR-Alus (SVAs) [78,79,80]. At least 13 SVA insertions causing the disease have been identified so far, including one that causes X-linked dystonia Parkinsonism (XDP) [81,82]. Moreover, SVAs have the capability to influence tissue-specific gene expressions [83]. Using the whole genome sequence and transcriptomic and clinical data from the Parkinson’s Progression Markers Initiative (PPMI), the researchers identified eighty-one SVAs polymorphic for their presence/absence, seven of which were linked with PD progression [84]. Ongoing mobilization of SVAs has led to the emergence of polymorphic insertions known as retrotransposon insertion polymorphisms (RIPs) [81,85]. One of these RIPs, SVA-67, is located 12 kb upstream of the KANSL1 (KAT8 regulatory non-specific lethal complex subunit 1) gene, which is part of the MAPT locus (microtubule-associated protein tau). MAPT is considered as a significant risk factor for neurodegenerative diseases, such as AD and PD [86,87,88,89]. It has been demonstrated that SVA insertions have the potential to influence the expression of multiple genes over large distances and that regulation can be isoform-specific [80]. SVAs could influence the disease course of PD and ALS through modulation of isoform expression and usage, which ultimately could affect protein levels and biological processes. Environmental exposures to pesticides, such as rotenone, paraquat, dieldrin, and lindane, can also lead to the onset of PD [90,91,92,93]. Head trauma through direct or indirect blows caused by sports injuries, vehicle accidents, falls, etc., can also contribute to the onset of PD [94,95]. These findings indicate complex gene–environment and gene–gene interactions in neurodegenerative diseases, which is still an underexplored area.

## 4. Lewy Bodies in Dementia and Aging

Dementia with Lewy body (DLB) is a disorder that is associated with age as a risk factor. Lewy bodies (LBs), which are abnormal deposits of a protein called α-syn, are a hallmark of PD and are present in most DLB patients [96,97]. The most common symptoms of DLB are changes in cognition, movement, and sleep disturbance, which can be easily associated with PD symptoms [98]. Other signs and symptoms, such as a reduced sense of smell, constipation, frequent falls, and apathy, are commonly observed in both PD and DLB [99].

Neurodegenerative diseases, such as Alzheimer’s disease (AD) and DLB, are accompanied by structural changes in the brain that lead to functional changes. The major pathological difference between AD and DLB detected by imaging is that medial temporal lobe atrophy is less severe in patients with DLB when compared to AD [29,100]. Gray matter density (GMD) decreases nonlinearly with age, especially between 7 and 60 years old. This is most noticeable in the dorsal frontal and parietal association cortices on both the lateral and interhemispheric surfaces [101], suggesting progressive age-associated neuronal loss. Microglia are the resident macrophages of the CNS and are the primary immune effector cells in the brain. Microglia homeostasis is regulated by intrinsic and extrinsic factors [102,103]. Aging leads to a decline in microglial phagocytic activity [104,105,106], which may cause the accumulation of the misfolded α-syn protein. This α-syn protein aggregates over time and leads to the formation of LBs, as described in Figure 1. Microglia in the aging brain are believed to be “primed” but do not necessarily enhance the brain’s response to immune challenges. By contrast, peripheral immune challenge may induce robust inflammatory responses [107,108]. α-syn aggregation activates microglia and may lead to a cycle of neuroinflammation [109]. Like PD, mitochondrial dysfunction can also cause oxidative stress and chronic inflammation, which are contributing factors in aging-associated degenerative changes in the brain [110,111].

## 5. Calpain’s Role in Neuroinflammation and PD

Calpain, an intracellular non-lysosomal neutral protease, is involved in different cellular processes, including neuronal remodeling, axon degeneration, apoptosis, cellular proliferation, and cell motility [27,112]. The excessive activation of calpain influences several pathways, such as neuroinflammation, ROS, apoptosis, and autophagy [21,24,113,114]. Calpain over-activation is observed in various neurological disorders and injuries such as PD, AD, muscular dystrophy, traumatic brain injury, and spinal cord injury. Its activity increases with mutated α-syn [115]. Activation of the RhoA pathway plays a crucial role in cell signaling in both neurons and glial cells [116,117,118]. The Rho-ROCK pathway regulates various cellular processes, including cytoskeleton reorganization, cell death, mitochondrial homeostasis, autophagy, inflammation, and gene transcription.

Calpain consists of two subunits, a large subunit containing a cysteine protease and a small subunit containing a calcium binding site. There are a number of calpain isoforms, but calpain-1 and calpain-2 are ubiquitously expressed in the CNS. Calpain-1 is activated by micromolar (µ) calcium levels, while calpain-2 is activated by millimolar (*m*) calcium levels [27]. Once activated, calpain is thought to cleave α-syn. This cleaved α-syn activates toll-like receptors 2 and 4, causing inflammatory signal activation and oxidative stress in dopaminergic neurons [61,119]. Toll-like receptor and α-syn interact with CD36, which contributes to additional neuroinflammation and neurodegeneration. PD has a direct impact on calcium homeostasis [120,121]. During PD, or the introduction of neurotoxin, oxidative phosphorylation is disrupted, which occurs with an impaired electron transport chain in mitochondria, leading to inhibition of Complex I, reduced production of ATP, and increased calcium concentrations [121,122,123]. Since calpain is activated by calcium, increased levels of calcium lead to increased levels of activated calpain. Calpain cleaves the sodium exchanger in the electron transport chains Complex I and impacts Bax-2 and Bcl-2, and they determine the fate of cell survival. Additionally, studies have reported that calpain could be linked to the deregulation of BDNF signaling, causing synaptic dysfunction [124].

### Calpain-1 vs. Calpain-2 in Neuroinflammation and PD

Both calpain-1 and calpain-2 are ubiquitous and uniformly distributed in neurons and glia [124,125]. Each plays a specific role in the brain. Calpain-1 has been found to be neuroprotective, whereas calpain-2 is known to be neurodegenerative [21]. Our laboratory has recently shown that both calpain-1 and calpain-2 expression increased in SN dopaminergic neurons in a rotenone rat model of PD [21]. Although rotenone administration induced over-expression of calpain-1 and calpain-2 in the rat brain, calpain-2 appeared to be more degenerative, as its expression correlated with the death of dopaminergic neurons in the SN. Interestingly, blocking calpain activation, especially calpain-2, decreased neuronal loss in rotenone-injected rats [21]. This finding suggested that activation of calpain-1 and calpain-2 occurs in rotenone rats, but inhibition of calpain-2 could be more important to attenuate degenerative events in PD.

Calpain-2 is expressed in many different tissues in mammals, and unlike other proteases, calpain-2 does not break down its target proteins completely [126]. Instead, it cleaves them into smaller pieces that have different functions, are distributed differently, and interact with other proteins in different ways [127]. The many different activities associated with calpain-2 are a result of its ability to produce these different protein fragments. Calpain-2 is shown to be involved in the breakdown of α-syn in PD and huntingtin protein in Huntington’s disease (HD), and it cleaves TDP43 proteins implicated in motor neuron function in Amyotrophic Lateral Sclerosis (ALS) [96,128,129]. Because of this distinct function, calpain-2 could be seen as a central player in a variety of signaling pathways.

Calpain-2 could become hyperactive in the later stages of neurodegenerative diseases such as AD. This hyperactivity may increase beta-amyloid deposits, which can lead to further cognitive decline [130,131]. Overall, calpain-1 and calpain-2 play important roles in organizing neurotransmitter receptors, releasing neurotransmitters, regulating cytoskeletal dynamics, and facilitating local protein translation [27,132,133,134].

## 6. Neurotoxicity in PD and Aging

The pathophysiology of neurodegenerative diseases is very complex and still needs to be fully understood. Animal models created using different neurotoxin targeting SN dopaminergic neurons provided a valuable tool to investigate the disease process [135,136,137]. The most popular rodent models to study PD are generated by using different treatment paradigms of neurotoxins in mice or rats. The PD models are induced by i.p. injections of MPTP (1-methyl-4-phenyl-1,2,3,6-tetrahydropyridine) in mice, s.c. injections of rotenone in rats or mice, and lesion of dorsal striatum by intracerebral infusion of 6-OHDA in rats [24]. These toxin-induced models imitate PD pathogenesis by replicating the degeneration of dopaminergic neurons in the SN. These toxins primarily disrupt complex I of the mitochondrial electron transport chain, affecting cellular processes such as oxidative phosphorylation, adenosine triphosphate (ATP) depletion, reactive oxygen species (ROS) production, and elevation of intracellular Ca^2+^ levels [114,138,139]. By replicating the functional circuitry dysfunction of the basal ganglia caused by the death of dopaminergic neurons in the SN, these neurotoxin models provide valuable information about the cellular and molecular events occurring during the disease process [21,24,26,61].

While MPTP is specific to PD induction in mice, MPP^+^ is a metabolite of MPTP that is used in in vitro studies to test disease mechanism to gain insights into the pathogenesis of PD. Rotenone, 6-OHDA, and paraquat are used in both mice and rats to induce PD-like diseases [135]. These neurotoxins activate microglia and astrocytes, which lead to the release of cytokines, chemokines, and free radicals, just as in naturally occurring PD [19,140,141,142]. This leads to microglial activation and increased expression of MHC-II proteins that can interact with CD4+ T cells, thereby promoting T-cell infiltration and inflammation [9,24,143]. There are pro-inflammatory and anti-inflammatory microglia functions [140,144]. PD causes the activation of more pro-inflammatory microglia, promoting inflammation and neurodegeneration [142]. The following inflammatory markers have been found as markers of PD: MHC-II, IFN-γ, and TNF-α. Each of these factors further activates microglia and astrocytes. Investigating these neurotoxin-induced changes should provide a valuable tool for designing and generating new animal models to gain a better understanding of this neurodegenerative disease and development of therapeutic interventions. Most PD cases are believed to be sporadic, and various environmental factors may affect the risk of developing the disease. Pesticide exposure, genetic susceptibility, and traumatic brain injury increase the risk, while tobacco smoking and physical activity are known to be protective factors [145,146]. It appears that the interplay among aging, genetics, and environmental factors may be involved in the onset and progression of PD. Despite evidence that aging is a primary risk factor for PD, the biological connection is still unclear.

The significance of aging in the development of PD (in most cases) highlights the importance of developing animal and in vitro models of PD. Analyzing the findings from these models may help gain insights into the cellular and molecular events that occur during the progression of the disease. As epidemiological studies have strongly linked aging with the development of PD, there must be more shared biological pathways between the two, suggesting that age-related changes may lead the way for the dopaminergic neurodegeneration observed in PD. The number of SN neurons declines by 7 to 9.8% per decade in healthy individuals with normal aging [147,148]. Dopaminergic neuronal populations in SN are vulnerable to loss with aging compared to other brain regions, e.g., the hippocampus. SN neurons are particularly susceptible to mitochondrial dysfunction, which accumulates within them with advancing age. These changes could be important to the pathogenesis of PD.

The accidental discovery of neurotoxin MPTP gave an experimental tool to investigate PD pathogenesis in animal models and demonstrated the critical role of mitochondria in the disease process. This toxin and other pesticides, such as paraquat and rotenone, recapitulated PD and showed that the SN cell loss occurred via inhibition of complex I of the electron transport chain in mitochondria [149,150]. These findings were supported by reports of reduced complex I activity and protein expression in brain tissues from patients with PD [151,152].

A close relation between an age-related reduction in nigral TH neurons and an increased intracellular α-syn accumulation strongly suggests that aging represents a subthreshold pre-Parkinsonian state [153]. The aggregation of misfolded proteins in neurons is related to oxidative stress and neuroinflammation, and possibly, some unknown factors caused by reactive oxygen and nitrogen species, which accumulate within the SN with advancing age. These extrinsic and intrinsic events in the brain predispose the SN neurons to be under significant oxidative stress with a high concentration of damaged proteins, suggesting that efficient protein degradation pathways are critical for cellular integrity and function.

## 7. Activation of Rho-ROCK Pathway in PD and Aging

**Figure 2:**
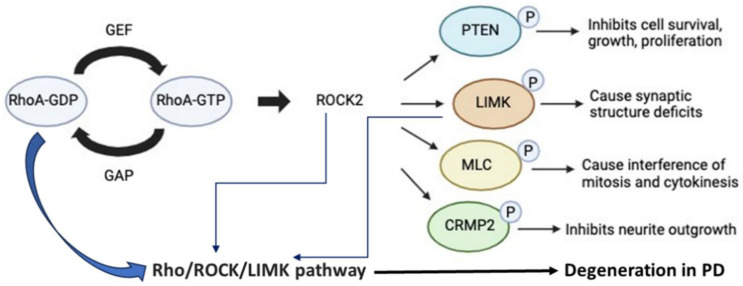
Activation of the Rho-ROCK pathway triggers PTEN, LIMK, MLC, and CRMP2 molecules and related pathways, influencing cell survival and death. The activation of Rho/ROCK/LIMK pathway also leads to neurodegeneration in PD. Arrows indicate various steps in the neurodegeneration process.

Rho GTPases regulate cytoskeletal and cell adhesion dynamics [154,155,156]. They are involved in cell morphogenesis, cell survival, cell proliferation, and cell migration (Figure 2). Rho GTPases are also molecular switches that cycle between an active GTP-bound state and inactive GDP-bound state. This switch is controlled by guanine nucleotide-exchange factors (GEFs) that exchange between GTP, GDP, and GTPase activating proteins (GAPs) [157,158]. The Rho GTPases are inhibited by catalyzing GTP hydrolysis. When Rho is GTP-bound, it interacts with downstream effector proteins, such as Rho-associated Kinase-1 (ROCK1), Rho-associated Kinase-2 (ROCK2), and DIAPH1 [154,158]. ROCK is a serine-threonine kinase that promotes actomyosin contractile force generation [159,160,161]. ROCK does this by increasing myosin light chain, a subunit of motor protein myosin II, phosphorylation. While there are multiple types of ROCK [162], ROCK2 is primarily associated with PD. ROCK2 is found in the brain and spinal cord, whereas its isoform, ROCK1, is found in non-neuronal tissue, such as glia [163]. The high RhoA activity in the cells can cause ROCK activation and upregulate the activity of phosphatase and tensin homolog (PTEN). Activation of the Rho-ROCK pathway stimulates protein/lipid phosphatase and tensin homolog, which inhibits cell growth and survival (Figure 2).

RhoA-ROCK signaling induces LIM kinase-dependent phosphorylation and inactivates cofilin, a mediator of actin turnover involved in disassembling actin filaments [164]. This leads to the buildup of filamentous actin (F-actin) and the formation of actin stress fibers, significantly influencing the dynamics of the cytoskeleton, as described by Villalonga et al. [165]. The phosphorylation of MLC initiates the interaction between actin and myosin, leading to heightened contractility of the actin cytoskeleton [166]. CRMP2 plays a role in regulating various aspects such as neuronal polarity, axon guidance, dendritic projection, and the migration of neurons and immune cells. Hyperphosphorylation of CRMP2 is commonly linked to neuronal injury and degeneration [167,168,169]. Although RhoA-ROCK signaling induces neuroinflammation and neurodegeneration, it also activates collapsin response mediator protein-2, which is involved in stimulating the promotion axon growth with microtubule assembly [154,167].

### 7.1. Activation of Rho-ROCK Pathway in PD

The activation of the Rho-ROCK pathway is associated with microglial activation and astrocyte activation. Microglia are immune cells of the CNS. Microglia aid in controlling α-syn levels, so when microglia are impaired, dysregulated α-syn increases along with LB aggregates [170,171,172]. The activation of these cells leads to neuroinflammation and neurodegeneration through the release of inflammatory cytokines and chemokines. The cell-to-cell transfer of α-syn also contributes to the development of PD, suggesting the spread of α-syn pathology [172]. The spread of extracellular α-syn can also disrupt microglial autophagy activity, contributing to neuroinflammation and PD development.

Inflammation and activation of ROCK are associated with loss of function in α-syn clearance mechanisms in microglia. Activation of the Rho-ROCK pathway also leads to disruption of the blood–brain barrier, which allows infiltration of immune cells and harmful substances in the brain. The activation of Rho-ROCK signaling not only leads to neuroinflammation, but it also alters the autophagy process, which can aid in the accumulation of damaged proteins, a contributing factor in neurodegeneration. Inhibiting the ROCK pathway could be a way to design therapy to prevent worsening neurodegeneration and inflammation in the PD brain.

### 7.2. Activation of Rho-ROCK Pathway in Aging

**Figure 3:**
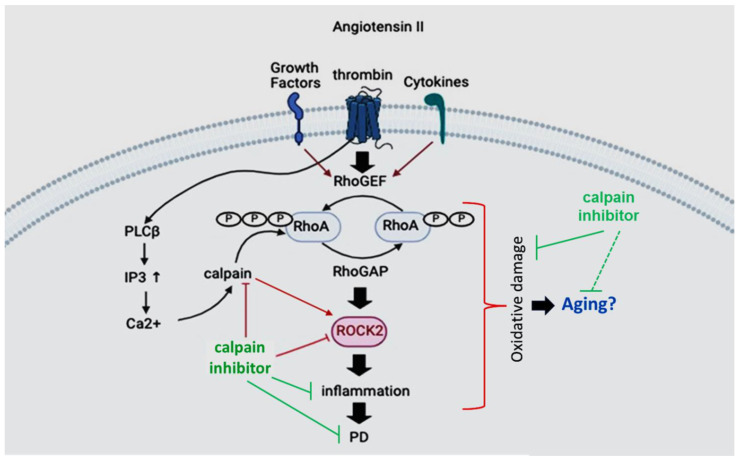
This figure displays the upstream and downstream components of the Rho-ROCK pathway, and how angiotensin II and calpain are thought to be involved in regulation of Rho-ROCK function in inducing oxidative damage in PD and aging. Inhibition of calpain may help reduce Rho-ROCK activation and neuroinflammation, attenuating neurodegeneration in both PD and aging. Arrows indicate critical steps in the induction of inflammation and neurodegeneration in PD.

Abnormal activation of the RhoA/Rho pathway may contribute to the induction of neuroinflammatory and pro-oxidative responses, axonal retraction, and apoptosis. Increased expression of RhoA and ROCK II proteins and ROCK activity have been shown in the brains of aged rats, particularly in the substantia nigra [173]. Increased ROCK activity may enhance major mechanisms responsible for aging-related neurodegeneration, thus representing a major factor in the vulnerability of dopaminergic neurons to damage. Thus, inhibition of ROCK2 may constitute an effective neuroprotective strategy against aging-related risk of dopaminergic degeneration and possibly against other aging-related neurodegenerative processes (Figure 3).

A recent study tested the role of RhoA-ROCK-mediated Wnt/β-catenin signaling in the regulation of aging-associated disorders [174]. This study found that high ROCK activity closely correlated with Jak and Gsk3β activities but inversely correlated with β-catenin signaling activity in bone marrow mesenchymal stromal cells from elderly male humans and mice. Another study tested soleus feed arteries (SFAs) from young (4 months) and old (24 months) male Fischer 344 rats [175]. In this study, smooth muscle cells isolated from one group of SFA were assessed for phosphorylated ROCK. Interestingly, total ROCK1 and ROCK2 were similar in cells isolated from young and old SFAs, whereas phospho-ROCK1 and phospho-ROCK2 levels were higher in cells isolated from old SFAs relative to young arteries. These results suggested that smooth muscle contractile function declines with age in SFA. Thus, the study of the Rho-ROCK pathway in aging and neurodegenerative diseases (e.g., PD and AD) should be investigated further in relevant animal models and humans.

## 8. Calpain Activation and Regulation of Rho-ROCK Signaling in PD

Rho GTPases have a particular importance in the CNS: developing neurons, neuronal survival, growth, axon and dendrite branching, and forming and maintaining dendritic spines [154,176,177]. Rho GTPases are essential to maintain plasticity of synapses. When Rho GTPases are dysregulated, many neurological disorders and diseases can take place, including schizophrenia, depression, ALS, autism spectrum disorders, PD, and AD. RhoA, however, acts as an inhibitor of these processes: promoting neuronal death, retraction of axonal and dendritic spines, and hence synapse loss. RhoA has been found to be elevated following injury to the brain in both animals and humans. This elevation of RhoA can restrict regeneration and full functional recovery in the CNS. An increased activity of RhoA in PD patients may increase phosphorylated α-syn, and therefore, both calpain-1 and calpain-2 isoforms could be activated in the nigrostriatal pathway. Activated calpain may also stimulate the ROCK pathway, leading to the cleavage of IkB [143,178], which causes NF-kB to translocate to the nucleus. NF-kB is crucial in biological responses. NF-kB controls cell growth, proliferation, apoptosis, cell survival, stress response, etc. [179]. Activated NF-kB also leads to inflammation, which causes death to surrounding dopaminergic neurons. The death of these dopaminergic neurons leads to loss of motor function.

RhoA-ROCK signaling in microglia is thought to be a major player in the progression of neurodegenerative disorders [180]. Although ROCK is ubiquitously expressed in all tissues, ROCK2 subtype expression in the brain and the spinal cord is more abundant and improves with age, triggering inflammation. ROCK2 is also thought to be involved in angiotensin II-induced inflammation, and attenuation of calpain expression and activity may decrease ROCK2 and attenuate inflammation (Figure 3). Angiotensin I and angiotensin II receptors are in dopaminergic neurons, nigral microglia, and astrocytes [181,182,183]. These receptors are in the angiotensin pathway, and the activation of angiotensin I and angiotensin II speeds microglial receptor NADPH oxidase and glial inflammatory response. Angiotensin I receptors also mediate the Rho-ROCK pathway and microglial activation. Thus, inhibiting ROCK could significantly decrease microglial activation and dopaminergic cell death [184].

## 9. Targeting Rho-ROCK Pathway in PD and Aging

Activation of RhoA is the beginning of the ROCK pathway. Activation of the ROCK pathway in PD causes axonal growth inhibition, loss of dendritic spine plasticity, inhibition of Parkin mitophagy, inhibition of autophagy, inhibition of apoptosis, inhibition of cell division, inhibition of cell contractility, and inhibition of cell mobility [185,186]. Inhibiting the ROCK pathway is a promising therapy for improving PD-caused damage in the body. There are multiple ROCK pathway inhibitors: fasudil, Y-27632, statin, onjisaponin B, loganin, and KD025 [187,188,189,190]. Fasudil has been used in neuroblastoma cell lines, mice, rats, H4 cell culture, and mesencephalic culture [191]. Fasudil has been found to improve neurite outgrowth, lower expression of ROCK2, and decrease dopaminergic neuron loss in neuroblastoma cell lines.

Fasudil has been found to lower α-syn aggregation in H4 cell culture. In mice, fasudil improved motor and cognitive function and increased DOPAC, as well as decreased RhoA and ROCK2 mRNA levels and ROCK activity in microglia. Fasudil also improved motor function when given to rats with induced PD [192]. Y-27632 has been found to lower dopaminergic neuronal loss in neuron glia culture, increase dopaminergic neurons and grafted dopaminergic fibers along with lessened behavioral impairments in rats, improved neuroprotective effects and removal of damaged mitochondria in drosophila melanogaster, and in SH-SY5Y neuroblastoma cells [193]. Y-27632 administration lowered apoptosis through inhibition of ROS generation. Statin lowers α-syn accumulation in mice and neuroblastoma cells and protects dopaminergic neurons from damage in mesencephalic cell cultures. Onjisaponin B treatment in mice lowered the expression of RhoA and ROCK2, lessened dopaminergic degeneration, and reduced microglia activation and inflammatory factor expression. Loganin in PC12 cells increased Akt and GSK-3beta signaling pathway, increased Nrf2/HO-1 signaling pathway, lowered ROS levels, lowered apoptosis, and lowered mitochondrial membrane permeability. In mesencephalic neuronal culture, loganin administration inhibited the RhoA-ROCK pathway. In mice, loganin administration lowered ROS levels and MPTP toxicity [194].

## 10. Conclusions

PD involves a complex array of symptoms, including the motor symptoms in the SN. The activation of calpain, α-syn aggregation, and oxidative damage may promote the Rho-ROCK pathway, inducing neuroinflammation and neurodegeneration in PD and other forms of dementia. Inhibiting calpain to reduce inflammation and protect motor neurons from degradation could be a possible therapeutic strategy. Inhibiting calpain could also reduce glial activation, which would help prevent dopaminergic neuron loss in the substantia nigra. Preventing dopaminergic neuron loss would slow the loss of dopamine required for motor function, allowing PD patients to have a slower, or lessened, loss of motor function. Calpain inhibition could also attenuate Rho-ROCK activation and reduce oxidative stress in aging. Overall, regulating the Rho-ROCK pathway could be a promising therapeutic strategy to lower dopaminergic neuron loss, improving the quality of life of those with PD. In addition, regulating this Rho-ROCK pathway could not only benefit individuals with PD, but also those with hypertension, angina, vasospasm, atherosclerosis, stroke, heart failure, coronary vasospasm, endothelial dysfunction, AD, HD, and ALS, as Rho-ROCK is implicated in these disorders. Since dopaminergic neuronal populations seem vulnerable to loss with aging as compared to many other brain regions, a reduction in calpain and Rho-ROCK activation could also benefit neurodegenerative disorders in aging.
